# Mothers’ and Fathers’ Worry and Over-Control: One Step Closer to Understanding Early Adolescent Social Anxiety

**DOI:** 10.1007/s10578-018-0807-7

**Published:** 2018-05-05

**Authors:** Nejra Van Zalk, Maria Tillfors, Kari Trost

**Affiliations:** 1 Department of Psychology, Social Work and Counselling, University of Greenwich, London, UK; 2 Department of Social and Psychological Studies, Karlstad University, Karlstad, Sweden; 3 Department of Child and Youth Studies, Stockholm University, Stockholm, Sweden

**Keywords:** Social anxiety, Parental worry, Parental over-control, Emotion regulation, Early adolescence

## Abstract

This study investigated the links between parental worry, parental over-control and adolescent social anxiety in parent-adolescent dyads. Using a longitudinal sample of adolescents (*M*_age_ = 14.28) and their parents (224 mother–daughter, 234 mother–son, 51 father–daughter, and 47 father–son dyads), comparisons were conducted using cross-lagged path models across two time points. We used adolescent reports of social anxiety and feelings of being overly controlled by parents, and mother and father self-reports of worries. Our results show that boys’ social anxiety predicted higher perceived parental overcontrol, whereas girls’ social anxiety predicted higher paternal worry over time. In addition, girls’ reports of feeling overly controlled by parents predicted higher maternal worry but lower paternal worry over time. For boys, feeling overly controlled predicted less social anxiety instead. The study illustrates how mothers and fathers might differ in their behaviors and concerns regarding their children’s social anxiety and feelings of overcontrol.

## Introduction

The relationship between children’s social anxiety and perceived parental over-control has been well established in previous literature (for extensive reviews, see [1–3]). Jointly, findings point to evidence of a direct link between perceived parental behaviors relating to over-control on the one hand, and children’s anxiety on the other. Characterized by parental overinvolvement, over-control of anxious children is believed to stem from parental attempts to protect them from potential distress [1–3]. Although the intention might be good, by taking over responsibilities which children might well be capable of doing independently, parents might inadvertently encourage them to become overly dependent or take on avoidant behaviors that lead to lack of autonomy or fears in social contexts [1–4]. Shielding children from new, potentially stressful experiences, although helpful in the short term, might help set them up for social failures later on.

Indeed, being exposed to novel situations might be more meaningful than expected, as they allow children to experience harmless mistakes and limited discomfort. Nevertheless, parents might contribute to a social environment where children don’t get exposed to novelty to a large degree. Parental over-control of children’s everyday activities is believed to limit children’s experience of novel situations [2, 5], which might be particularly important for the development of *social anxiety*, as it involves discomfort and inhibition predominantly in new social situations and contexts [6]. Social anxiety is characterized by social fears, excessive discomfort, worry, rumination, and somatic symptoms such as trembling, blushing and sweating before, during, and after social interactions [7]. By restraining opportunities for their children to become more effective in new social settings, parents might unintentionally expedite their socially anxious children towards increased social failures. By hindering their child from practicing social behaviors in novel situations, parents could limit the potential for the child to become confident, comfortable, and less anxious around others.

The links between parental over-control and social fears have been identified in childhood [8–11] as well as early adolescence [12, 13], confirming the notion that children’s social anxiety might be affected by, and might in turn affect, parental controlling behaviors. The reason behind parents’ restriction of their socially anxious children’s autonomy development might be linked to worries about their children, however. Worrying, or focusing one’s attention on possible future threats, is a common emotion regulation strategy that might lead to increased anxiety [14]. Some parents might start worrying about their socially anxious children’s well-being, which could prompt over-controlling behaviors—and might subsequently be modelled by the children. By overly worrying, parents might inadvertently be mentoring their children, thus increasing the children’s social anxiety over time. Indeed, socially anxious parents tend to have socially anxious children [15, 16]. By modelling their parents, scholars have suggested that anxious children learn that the world is a dangerous place where they might expect negative consequences for their behavior and become increasingly fearful and avoidant as a result [17]. Hence, parental worries might send signals that in turn help reinforce children’s social fears over time, but more research is needed.

Another aspect that might influence the relationship between social anxiety and parental over-control is parents’ gender. Scholars have postulated that fathers play a more important role than mothers in the development of social exploration and autonomy [18]. Energetic, loud play with children, often conducted by fathers as opposed to mothers, might be more prominent in father–child interactions—even though mothers have been found to play with their children just as much [19]. These types of interactions might make fathers especially important in the eyes of their children. Studies looking at father involvement indicate that children with fathers who are involved in their lives show increased cognitive competence, and scholars have posited that this might be due to having more than one highly involved parent [18]. Another way fathers might influence socially anxious children particularly is through displayed attitudes and behaviors. For instance, research indicates that fathers tend to use more straightforward, commanding language, with more requests for clarification than mothers (e.g., [20, 21]). Through the use of imperative language, fathers are believed to teach their children about the communicative demands of social interactions [18]. Related to this, school-age children’s perceived quality of the father–child relationship is associated with social competence and peer acceptance [22]. Along similar lines, Bögels and Phares [23] found that fathers are important in encouraging independence and social behaviors, whereas Fliek et al. [24] report that mothers are important for emotional support. Verhoeven et al. [25] report that mothers’ over-control was linked to childhood anxiety but father’s over-control was more important for perpetuating adolescent anxiety. In one longitudinal study focusing on young boys’ behavioral inhibition, a precursor to social anxiety, the results indicated that fathers’ but not mothers’ intrusive and less sensitive parenting predicted less inhibition for boys [26]. Despite these indications in the literature, research on how mothers and fathers, respectively, might affect the development of early adolescent social anxiety is sparse.

The majority of the current literature focusing on how parental treatment affects the development of social fears has examined mothers’ rather than fathers’ behaviors towards small children [27], however, with some notable exceptions. In one study, using vignettes describing ambiguous situations between parents and children, the authors found that if the father was described acting confidently or anxious influenced children’s state confidence or anxiety more than mothers’ actions for highly socially anxious children [28]. The findings are complex as children with low social anxiety rated their mothers as more influential [28]. The authors concluded that fathers might differ from mothers in their role of teaching social confidence to socially anxious children because mothers seemed to teach social wariness to children who were low on social anxiety [28]. That is, fathers might appear more confident and less anxious to youths with high social anxiety, thereby helping to reduce youths’ own anxiety. The participants were children aged 8–12 years old, and the data were cross-sectional, which precludes examining processes over time, and isn’t necessarily indicative of what happens during early adolescence. It is also important to note that the study did not examine parental worries. In another study, when controlling for paternal overcontrol, maternal overcontrol was found to indirectly impact the links between maternal and child anxiety, thus acting as a mediator [4]. Nevertheless, maternal anxiety rather than worries about their children were measured in the study.

In addition, little is known about the influence of adolescent-parent relationships in comparison with childhood. Empirical findings about parental overcontrol and adolescent anxiety are exceedingly scarce, because the existing studies tend to largely focus on either early childhood or adulthood [3]. Early adolescence in particular also appears to be a normative time of onset for social anxiety, as children start using cognitive emotion regulation strategies such as worry and rumination to a higher degree, and begin to experience increasing social demands [6]. In addition, young people’s roles and needs within the family change during early adolescence [29], and adolescents spend increasing amounts of time away from parents’ direct supervision [30]. Risky behaviors such as drinking alcohol or getting involved in delinquent activities typically start emerging during this time period [30], and might provide a genuine reason for parental worries. Experimentation with alcohol as well as involvement in illegal activities are higher within the adolescent population than among adults, though are unlikely to lead to alcohol problems or developing criminality later on [30, 31]. Yet these are types of activities that might cause genuine worries among all parents and might confound the links between parental worry in the area of adolescent social anxiety, where parents’ overcontrol is reportedly high. Thus, adolescent social anxiety and parental aspects that play a part in its development require more attention.

Finally, there are several important factors to take into account when examining links between parental worries and adolescent social anxiety. First, using information from multiple informants is important, as children typically report more problematic parenting than the parents themselves [32], with somewhat higher agreement within adolescent-parent relationships [33]. Because adolescent perceptions of parenting might have just as much impact on their development as actual parenting behaviors [34], adolescent reports might be more aligned with parent reports of their own behaviors. Furthermore, when comparing adolescent and parent reports of parental behaviors to those of external observers, adolescent reports appear to be more strongly related to what outsiders perceive in the parent–child interaction [35]. In particular, parental over-control reported by adolescents rather than by parents themselves might be the most valid in terms of consequences for their social anxiety. Second, and related to the above, adolescents’ reports regarding their own social anxiety might be more reliable than parents’ reports, as those who experience social fears do not always appear as socially fearful to others—especially familiar others [36]. With that noted, a similar logic might apply to parents’ reports of their own behaviors. Third, mothers tend to provide more positive impressions of their own parenting behaviors compared to children’s reports [37]. Relying on mothers’ reports alone might provide biased estimates of parenting. It is therefore important to include fathers’ as well as mothers’ reports of their own parenting behavior as each provide a unique perspective. Thus, including adolescent girls’ and boys’ reports of parents’ over-control and social anxiety, and information about worrying from their mothers and fathers, respectively, is likely to yield a more detailed picture of the dynamics under study.

In the current study, we used two waves of data from 982 early adolescents (52% boys; *M*_age_ = 14.28) and their parents (*N* = 859 parents; 467 mothers and 392 fathers) who participated in a community-based longitudinal study. In order to consider dyadic information sources, adolescent reports of social anxiety and parental over-controlling behaviors as well as parent reports of worry were obtained. To exclude the potential confounding effects of problem behaviors being viable reasons for worrying, we controlled for adolescent-reported problem behaviors at Time 1. Using a cross-lagged path model, we examined (a) the links between parent-rated worry, adolescent-rated parental over-control, and early adolescent social anxiety over time, and (b) if these links were moderated by parent and adolescent gender, respectively. Based on previous literature, we hypothesize the following: (1) there will be bidirectional links between parental over-control and worries and adolescent social anxiety; (2) mother–son, mother–daughter, father–son, and father–daughter dyads will differ on the links between parental over-control, worries, and social anxiety. Given the exploratory nature of research on parent-adolescent dyad differences, we did not develop more specific a priori hypotheses regarding these links.

## Method

The participants were from a community-based, cohort-sequential study in a city in Western Europe with a total population of about 26,000 inhabitants. The first of five annual data collections took place during the 2001-02 school year, with follow-ups in roughly 1-year intervals. At each wave, more than 90% of all adolescents in the community in grades 4 through 12 participated (approximately10–18 years old). All of their parents were invited to partake at every biannual wave (i.e., waves 1, 3, and 5), with participation rates at 68, 70, and 42% respectively across the three time points. Adolescent-reported social anxiety and parent reported worry were available at Waves 3 and 5. For simplicity, Waves 3 and 5 are referred to as Times 1 and 2. At Time 1, threats of unemployment (8.1%) and single-parent households (10%) in the community were similar to the national averages. Mean incomes were about 4% lower than national average.

Time-1 adolescent participants included 982 7th–9th graders (48% girls; *M*_age_ = 14.28) from 7 classrooms who were evenly distributed across 3 different schools. Approximately 10% of the adolescents in the sample were first-generation immigrants at Time 1. Sixty-six percent of the adolescents lived in households with both biological parents, 13% lived with one stepparent and one biological parent, 20% lived in single-parent households, and 1% lived with other relatives, foster parents, or in temporary foster care facilities. Time-1 parent participants included 467 mothers and 392 fathers (*N* = 859). Eighty-seven percent of the parents were born in the country, with 17% of the fathers and 10% of the mothers being university-educated. The proportion of missing values in the dataset was calculated using the covariance coverage matrix in MPlus, yielding an estimated proportion of all available observations for each variable used in the analyses [38]. The adolescents had between 36 and 99% of data available at both timepoints, whereas parents had between 43 and 86% data available at both timepoints. Because we only included participants with at least one wave of data, however, the final sample comprised 224 mother–daughter, 234 mother–son, 51 father–daughter, and 47 father–son dyads. As the group sizes indicate, mother participation was greater than for fathers.

## Procedure

Adolescents were recruited in classrooms during school hours. They were informed that participation was voluntary and their responses would be kept confidential. Information was provided about the questionnaire, including how long it would take to answer. Before adolescents were asked to participate, parents were informed about the study in meetings held in the community and by mail. Those who did not want their child invited to the study sent in a postage-paid postcard (1%). Parents were informed that their child could withdraw at any time throughout the study. Research assistants administered the questionnaires during regular school hours, and teachers were not present. Adolescents were not compensated monetarily for their participation in the study, but a drawing for movie tickets took place at the class level. Whether or not adolescents chose to participate, they were eligible for the drawings. Parents were sent questionnaires home along with a pre-addressed postage-paid envelope to return when they were finished. Only one parent per adolescent was asked to complete the questionnaires separately from the other parent. All of the procedures and measures were approved by the University’s Ethics Review Board.

## Measures

The measures in the current study were developed as part of a larger study focusing on a broad investigation of external and internal difficulties in adolescence, and the roles of parents, peers, and individual characteristics in the development of problem behaviors. Two strong emphases for the larger study were external adjustment (such as delinquency) and parenting. The aim of the study was to cover the broadest range of adolescent issues possible, including behavior at home, at school, and with peers. Besides for the measure of social anxiety, all of the other measures were piloted and developed for the project.

### Adolescent Ratings of Social Anxiety

Social anxiety was measured with 8 questions about fears in different social situations [39], which is a modified instrument based on the Social Phobia Screening Questionnaire adapted for adolescents [40]. The current version, the SPSQ-C (the Social Phobia Screening Questionnaire for Children; [41]), is comprised of two different parts. Only the first part was used in this study, which contains 8 social situations that tend to elicit social anxiety: “speaking in front of the class,” “going to a party,” “being with classmates during breaks,” “raising my hand during class,” “making a phone call to someone I do not know very well,” “initiating conversation with someone I do not know very well,” “eating with others during lunch,” and “looking in somebody’s eyes during a conversation.” Adolescents rated their fears on a three-point scale: *None* (1), *Some* (2), or *A lot* (3). The Cronbach’s *α’*s were 0.74 for Time 1 and 0.73 for Time 2. This measure has been validated using a diagnostic interview as a reference, with a sensitivity of 71% and a specificity of 86%. The instrument also has showed a moderate test–retest reliability, *r* = .60 [41].

### Adolescent Ratings of Feeling Overly Controlled by Parents

There were five items measuring whether youths felt overly controlled by their parents [42]. This scale comprises items similar to other measures of parental intrusive behaviors such as disrespecting children’s integrity and individuality (see e.g., [43]). The items were “Do you think your parents give you enough freedom to do what you want during your free time?” “Does it feel like your parents demand to know everything?” “Do you think your parents control everything in your life?” “Do you think your parents intrude into what you do in your free time?” and “Do you feel like you can’t keep anything to yourself, because your parents want to know everything?” The 5-point response scale ranged from *Yes, always* (1), *Yes, most of the time* (2), *Yes, sometimes* (3), *No, seldom* (4), to *No, never* (5). The items were re-coded so that 5 indicated always feeling controlled. The Cronbach’s *α* was 0.81 for Time 1 and 0.83 for Time 2. This measure has shown a high 2-month test—retest reliability in a previous study, *r*(36) = .82 [42].

### Parent Ratings of Worry About Adolescents

Parents responded to questions about worrying about their children [44]. The questions were “Are you worried that your child will not make it in school,” “Are you worried that the child will end up in bad company,” “Do you worry about what your child is doing together with friends on evenings and weekends,” “Are you worried that your child will start abusing alcohol,” “Are you worried that your child will start using narcotics,” and **“**Are you worried that your child will get caught by the police?” The response items ranged from *Yes, always* (1), *Yes, most of the time* (2), *Yes, sometimes* (3), *No, seldom* (4), to *No, never* (5). The items were re-coded so that 5 indicated always worried. The Cronbach’s *α*’s were 0.88 at Time 1 and 0.89 at Time 2.

### Adolescent Ratings of Problem Behaviors

We controlled for behaviors that might elicit real worry by parents, namely *delinquency* and *drinking*. These measures were used as control variables at Time 1 only. Delinquency was measured with 15 items pertaining to shoplifting, being caught by the police for something they had done, vandalizing public or private property, taking money from home, painting graffiti, breaking into a building, stealing from someone’s pocket or bag, buying or selling stolen goods, stealing a bike, stealing a car, stealing a moped or a motorcycle, stealing something from a car, doing anything that warrants being caught by the police, and being gone from school a whole day during the past year [42]. The response items ranged from *No, never* (1), *1 time* (2), *2–3 times* (3), *4–10 times* (4), to *More than 10 times* (5). The Cronbach’s *α* for delinquency was 0.84 for Time 1. Drinking was measured with a single item pertaining to the past year. The item was “Have you drunk so much beer, liquor, or wine that you got drunk,” and the response items were the same as for delinquency.

## Analyses

We conducted a cross-lagged path model using manifest variables in MPlus [38], with the FIML (Full Information Maximum Likelihood) procedure for all analyses. The use of the FIML procedure allowed for the recovery of the missing data for parents, as it makes use of all available data to estimate information about missingness in the dataset [45, 46]. By doing so, FIML provides less biased results than both pairwise and listwise deletion [45].

We included the following paths in the model: (a) stability paths between social anxiety, feeling overly controlled, and parental worry over time; (b) within-time covariation paths between social anxiety, feeling overly controlled, and parental worry at both time points; (c) cross-lagged paths between all variables; and (d) control paths from adolescent delinquency and drinking to parental worries at Time 1. To test for moderating effects of adolescent and parent gender, we employed multiple-group comparison procedures using χ^2^-difference tests. We used the initial cross-lagged path model to compare four dyad-based groups using parents’ and adolescent’s gender: mothers–boys, mothers–daughters, fathers–boys, and fathers–daughters. Model fit was evaluated using the Root Mean Square Error of Approximation [47] and the Comparative Fit Index [48]. In the current study, we use an RMSEA of less than 0.08 and a CFI greater than 0.95 to indicate an acceptable model fit. These cutoffs are based on accepted guidelines noting that RMSEA values of less than 0.08 represent an acceptable fit, whereas values less than 0.05 are considered a very good fit [47]. CFI values above 0.95 are considered acceptable fit, whereas values greater than 0.97 are considered good fit [48].

## Results

### What Are the Links Between Parent-Rated Worry, Adolescent-Reported Parental Over-Control, and Early Adolescent Social Anxiety Over Time?

Table 1 shows descriptives and correlations for all study variables. One-way ANOVAs comparing boys and girls revealed that girls had higher social anxiety and boys had higher levels of delinquency at Time 1, respectively, which would be expected (e.g., [49, 50]). In addition, mothers and fathers both reported slightly higher worries for boys than girls, and these were marginally significantly at Time 2 but not at Time 1. As Table 1 also indicates, social anxiety was positively associated with feeling overly controlled concurrently and across the two timepoints but was only negatively associated with drinking at Time 1. Mothers’ worries, on the other hand, were positively associated with feeling overly controlled as well as problem behaviors over time. Fathers’ worries were not significantly correlated with feeling overly controlled, but showed similar correlations with problem behaviors as mothers’ reports.

**Table 1:** Correlations and means (standard deviations) for all study variables with one-way ANOVAs for mean gender differences

Variables	1	2	3	4	5	6	7	8	9	Mean girls (SD)	Mean boys (SD)	F
Adolescent reports												
1. SA T1	–									1.40 (0.29)	1.32 (0.32)	6.31*
2. SA T2	0.48***	–								1.42 (0.32)	1.38 (0.33)	0.97
3. FOC T1	0.07*	0.11***	–							2.51 (0.78)	2.62 (0.89)	1.73
4. FOC T2	0.06†	0.04†	0.42***	–						2.28 (0.92)	2.36 (0.82)	1.19
5. Del T1	0.04	0.02	0.14***	0.04†	–					1.15 (0.28)	1.26 (0.46)	7.15*
6. Drink T1	− 0.06*	− 0.04	− 0.10***	− 0.15***	0.39***	–				1.75 (1.30)	1.77 (1.31)	0.03
Parent reports												
7. M.Wor. T1	− 0.03	0.02	0.19***	0.12*	0.32***	0.29***	–			1.88 (0.68)	1.98 (0.69)	2.26
8. M.Wor. T2	0.01	− 0.07	0.23***	0.25***	0.18**	0.18**	0.59***	n.a.	n.a.	1.79 (0.75)	1.89 (0.74)	3.51^†^
9. F.Wor. T1	− 0.17	− 0.11	− 0.03	0.25*	0.26*	0.35**	n.a.	n.a.	–	1.84 (0.89)	1.85 (0.89)	0.01
10. F.Wor. T2	0.06	− 0.12	− 0.08	0.05	0.34*	0.45**	n.a.	n.a.	0.67***	1.76 (0.83)	2.00 (0.84)	3.56^†^

*SA* social anxiety, *FOC* feeling overly controlled, *Del* delinquency, *Drink* drinking, *M.Wor*. mothers’ worries, *F.Wor*. fathers’ worries, *n.a*. not available as mothers’ and fathers’ reports weren’t available simultaneously, but rather either mothers or fathers reported about their worries at each time point

^†^*p* < .10; *p < .05; **p < .01; ***p < .001

To address our first research question, we examined the longitudinal links between adolescent social anxiety, adolescent-reported parental over-control, and parent-reported worry. We included stability paths and cross-lagged paths between social anxiety, feeling overly controlled, and parental worry from Time 1 to Time 2, and within-time covariation paths for both timepoints. In order to exclude the possibility that parents’ worries were justified, we included control paths from adolescent-reported delinquency and drinking to parental worry at both time points. As seen in the first column of Table 2, this initial model was acceptable (χ^2^ = 60.46; *df* = 8; *p* < .001; RMSEA = 0.08; CFI = 0.90). All paths were standardized. As the results indicate, there were no direct links between the variables, apart from stability paths for all constructs over time. Thus, our first hypothesis (that parental over-control and worries will predict the development of social anxiety in adolescents) was not supported.

**Table 2:** Standardized results for the main model and the multiple group comparisons

	ß all (*N* = 982)	ß mothers–boys (*N* = 224)	ß mothers–girls (*N* = 234)	ß fathers–boys (*N* = 51)	ß fathers–girls (*N* = 47)
Predicting social anxiety time 2					
Social anxiety T1	0.48***	0.35***	0.62***	0.52***	0.72***
Parental worry T1	0.02	0.08	− 0.03	0.05	− 0.12
Feeling overly controlled T1	0.05	0.09	0.07	− 0.25*	0.03
Predicting parental worry time 2					
Social anxiety T1	0.03	0.002	0.03	0.16	0.17*
Parental worry T1	0.59***	0.71***	0.39***	0.53***	0.61*
Feeling overly controlled T1	0.05	0.04	0.20***	0.28	− 0.13*
Delinquency T1	− 0.03	− 0.06	0.08	− 0.04	− 0.16
Drinking T1	0.08	− 0.13	0.16	− 0.32	0.55***
Predicting feeling overly controlled time 2					
Social anxiety T1	0.02	0.11^†^	0.00	0.03	− 0.004
Parental worry T1	0.04	− 0.05	0.08	0.08	0.08
Feeling overly controlled T1	0.47***	0.48***	0.42***	0.58***	0.33*
Within-time covariation paths					
T1 feeling overly controlled with worry	0.15***	0.09	0.19**	0.04	− 0.10
T1 feeling overly controlled with social anxiety	0.05	0.14*	0.05	0.01	− 0.07
T1 parental worry with social anxiety	− 0.001	0.01	0.01	− 0.12	− 0.14
T1 delinquency with parental worry	0.29***	0.33***	0.26***	0.33*	0.24
T1 drinking with parental worry	0.27***	0.32***	0.25***	0.25^†^	0.50**
T1 drinking with delinquency	0.53***	0.60***	0.58***	0.51***	0.50***
T2 feeling overly controlled with parental worry	0.11^†^	0.19^†^	0.23*	− 0.58**	0.18
T2 feeling overly controlled with social anxiety	0.04	0.01	0.08	− 0.24	− 0.50***
T2 parental worry with social anxiety	− 0.08	− 0.15^†^	− 0.10	0.33	0.25

^†^*p* < .10, *p < .05, **p < .01, ***p < .001

### Are These Links Moderated by Parent and Adolescent Gender?

In order to test for the moderating effects of parent and adolescent gender, we employed a multiple group comparison procedure using the same pathway model. The first step involved constraining all paths in the initial model to be equal between four groups, comprising mother–daughter (*N* = 224), mother–son (*N* = 234), father–daughter (*N* = 51), and father–son dyads (*N* = 47; χ^2^ = 168.21; *df* = 89; *p* < .0001). As a second step, we released the constraints for all paths in the model, allowing them to differ between the groups. We then used a χ^2^-difference test to examine the differences (χ^2^ = 55.81; *df* = 32; *p*_χ_^2^_−difference_ < .001). Because the test indicated that there were significant differences between the initial and the final models, the paths in the models were interpreted as moderating effects of parent and adolescent gender.

**Fig. 1:**
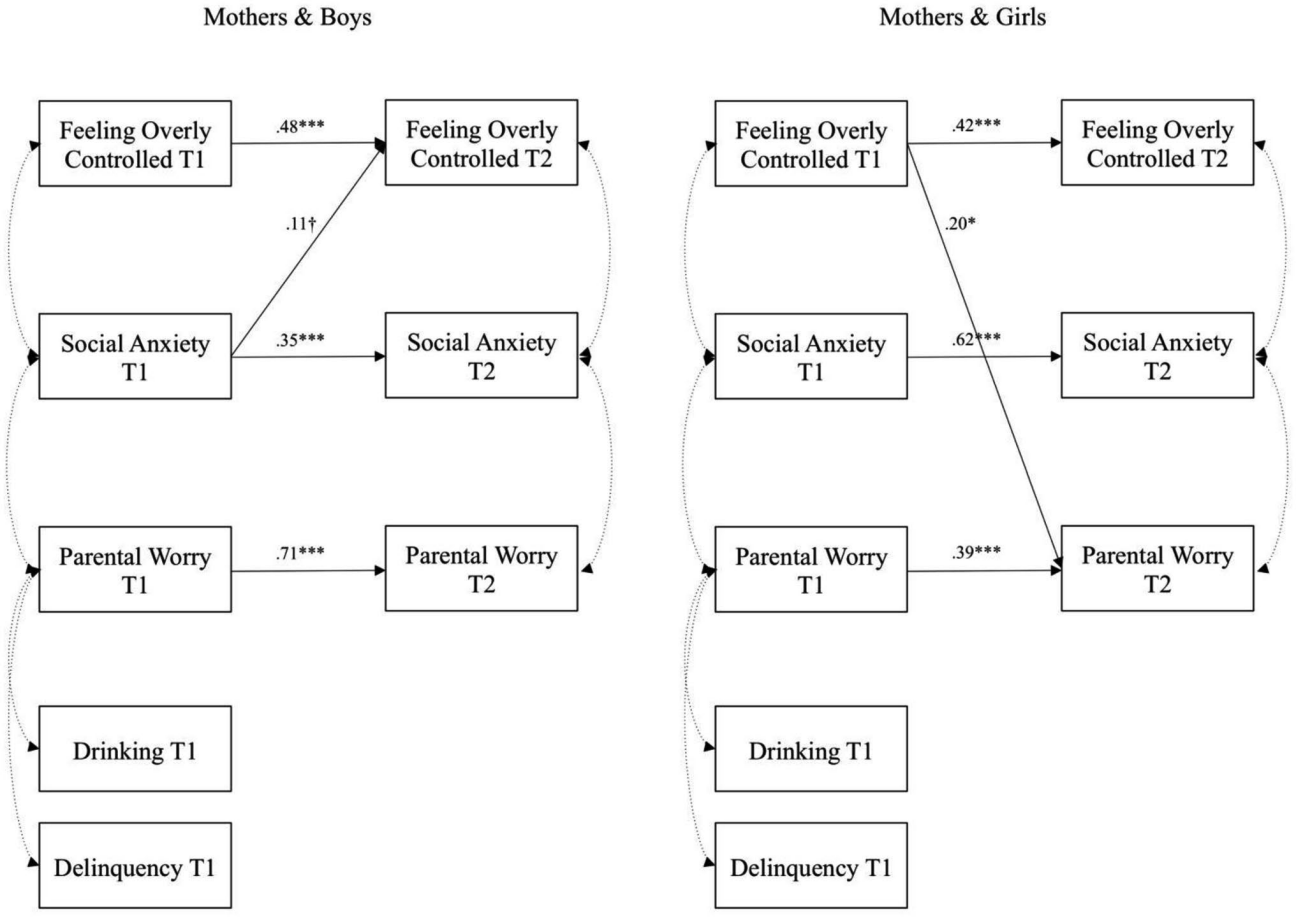
Results from multiple group comparisons depicting adolescents and their fathers. For the sake of clarity, non-significant paths are omitted from the figure. ^†^*p* < .10, *p < .05, **p < .01, ***p < .001

**Fig. 2:**
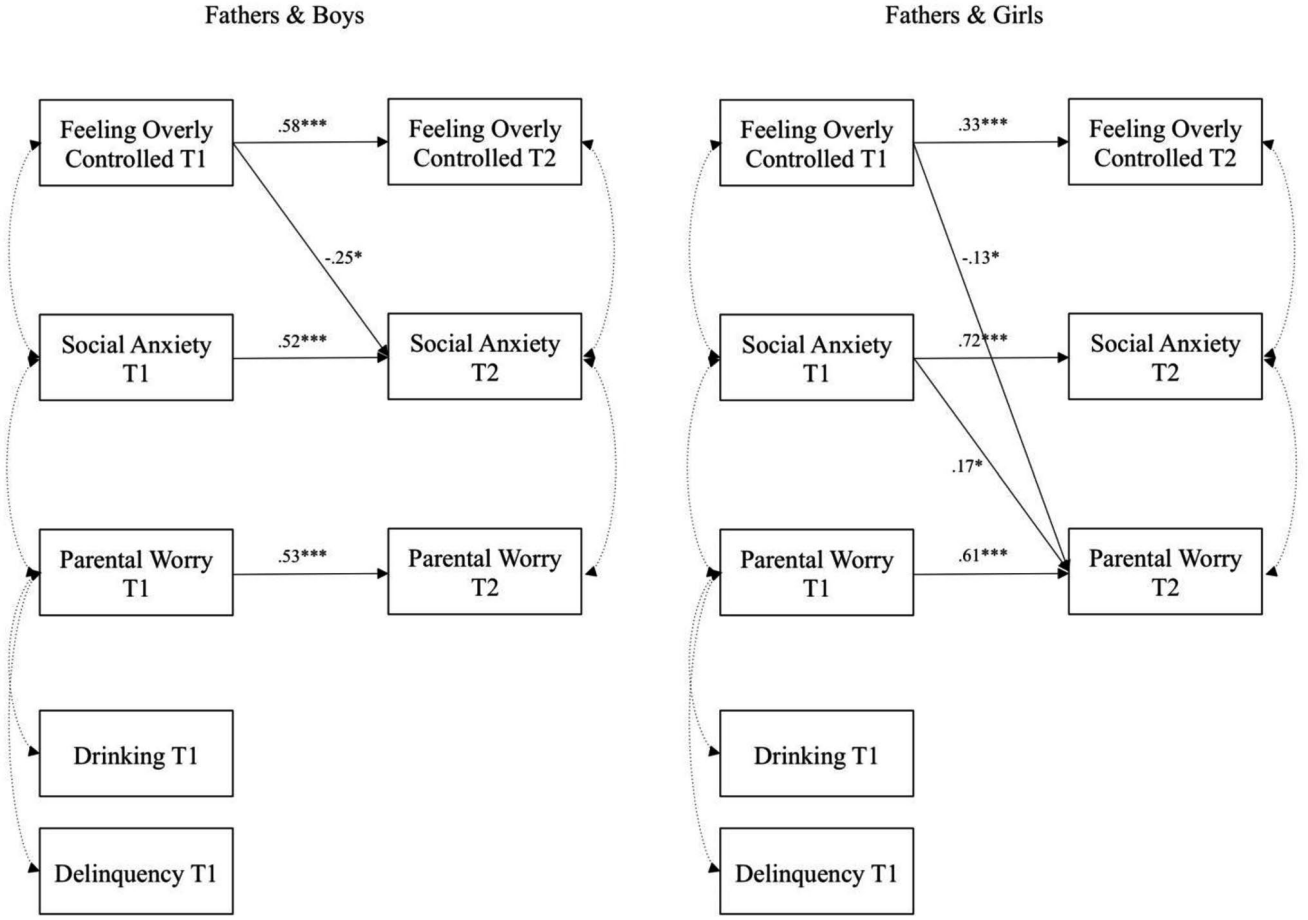
Results from multiple group comparisons depicting adolescents and their fathers. For the sake of clarity, non-significant paths are omitted from the figure. *p < .05, **p < .01, ***p < .001

The results are shown in Table 2 for each of the dyad groups separately, and depicted in Figs. 1 and 2, respectively. We found gender differences, as social anxiety was more stable over time for girls than for boys overall. Mothers showed significantly more stable worry for boys than for girls, whereas the opposite was true for fathers. Fathers reported more worry for girls than for boys. Nevertheless, the group difference tests show disparate pictures for the four dyad groups.

We hypothesized that there would be differences between the parent-adolescent dyads on the links between parental over-control, worries, and social anxiety, and the results support this hypothesis. For models with mothers, as depicted in Fig. 1, boys’ social anxiety at Time 1 predicted feeling overly controlled 2 years later, albeit marginally. For girls, however, feeling overly controlled at Time 1 predicted mother’s worries 2 years later. For father models, as shown in Fig. 2, feeling overly controlled predicted less social anxiety for boys and less parental worries for girls 2 years later. On the other hand, girls’ social anxiety predicted more paternal worries over time, whereas feeling overly controlled predicted less paternal worries. Thus, it seems that feeling overly controlled has the opposite effect regarding boys’ social anxiety in relation to their fathers compared to girls. This indicates that adolescents’ experience of parental overcontrol might have a buffering effect for boys’ social anxiety by reducing it over time, but the same links were not found for girls. These results also imply that early adolescent social anxiety and feeling overly controlled by parents has an impact on parental worries over time rather than the other way around. In addition, mothers and fathers appear to vary in their reactions to their children’s social anxiety and feelings of over-control.

## Discussion

Previous literature points to a strong association between parental over-controlling behaviors and children’s social anxiety (see reviews by [1–3]). Parents are believed get overinvolved in order protect their socially anxious children from prospective distress. Despite the best of intentions, however, parents might instead be adding to their adolescent’s anxiety over time [1–3]. Nevertheless, the current literature on parenting and adolescent social anxiety has been limited, as studies focus largely on small children as well as maternal behaviors only. In addition, there has been a lack of focus on parents’ worries about adolescents. In this study, we showed that mothers’ and fathers’ worries about their children, as reported by the parents themselves, were related to the development of early adolescent social anxiety in different ways—regardless of whether these worries were justified or not (i.e., by controlling for problem behaviors). We found that girls’ reports of feeling overly controlled by parents predicted more maternal worry over time. In the case of boys and mothers, it was boys’ social anxiety that predicted more feelings of overcontrol by parents over time, however. In models focusing on paternal worrying, feeling overly controlled by parents predicted less social anxiety for boys, and less paternal worry for girls. In addition, girls’ social anxiety predicted more paternal worrying across time. Jointly, these results indicate that mothers’ and fathers’ worries were not directly predictive of early adolescent social anxiety. Rather, it was either social anxiety or feeling over-controlled by parents that affected parental worries over time.

There is a small yet important literature concerning the differential roles that mothers and fathers play in the development of children’s anxiety. Nonetheless, the majority of this literature has examined mothers’ rather than fathers’ behaviors [27], with a few exceptions. One study found that fathers’ confident versus anxious behaviors influenced highly socially anxious children’s confident or anxious behaviors, respectively, more than mothers’ behaviors [28]. Intriguingly, mothers’ confident versus anxious behaviors were more influential than fathers’ behaviors for children with regular or low levels of social anxiety [28]. This indicates that fathers might teach social confidence to their socially anxious children, whereas mothers might instead be teaching social wariness to low socially anxious children [28]. To parallel the results in the current study, feeling overly controlled by parents predicted less social anxiety for boys in the models using fathers’ data. This might indicate that boys interpret fathers’ over-control differently compared to mothers, as fathers’ over-controlling behaviors might be associated with signals of assertiveness and confidence rather than restraint. Over-control, therefore, might not be as harmful in terms of social anxiety in father–son relationships. In addition, feeling overly controlled predicted less paternal worry for girls, which might indicate that fathers feel their over-control is justified and thus worry less. For mothers, on the other hand, the findings were somewhat different. Boys’ social anxiety predicted feeling overly controlled, whereas feeling overly controlled predicted more maternal worry for girls. This might suggest that for girls, mothers over-controlling behaviors create an environment that could enhance their worries. Mothers also showed more stable worries about boys compared to girls over time, whereas the opposite was true for fathers. Whatever the reasons for worrying—whether real or imagined—these findings need more probing in future studies.

Several scholars have suggested that temperamentally shy children might elicit different responses from parents [10, 51, 52]. When parents identify their children’s social insecurities, overcontrolling parental behaviors might consequently follow in order to attempt to help their child. The reverse might also be true over time, however, as overcontrolling parents could undermine children’s self-confidence. Children’s anxiety and parental over-control are likely to co-exist in a reciprocal pattern, and some evidence exists for bidirectional links between parents’ overcontrolling behaviors and adolescents’ social anxiety (e.g., [12]). The results from the current study indicate that boys’ social anxiety predicted feeling overly controlled by mothers, whereas feeling overly controlled predicted less social anxiety in father–son dyads. These results illustrate the importance of examining bidirectional processes over time and investigating variations within parent-adolescent dyadic processes.

This study has several limitations. First, there is a smaller number of fathers compared to mothers in this study, perhaps precluding the possibility to detect differences between the adolescent-parent groups—especially considering the sophisticated analyses used. Questionnaires were sent to adolescents’ homes and aimed at any of the parents, asking one parent per adolescent to fill it out separately from the other parent. It is perhaps stereotypical that mothers rather than fathers filled out the majority. There was also no way of controlling whether the parents actually filled out the questionnaires separately or together. In addition, the fathers that did choose to participate in the study might have contributed to selection bias, which might affect the external validity of the study. This is a limitation shared by many other studies, however, as fathers tend to be less included in research on clinical and pediatric problems in childhood compared to mothers [53]. Nevertheless, this is an important issue that needs to be combatted in future studies. Another limitation is that the adolescent ratings of feeling overly controlled by parents refer to both parents rather than one parent at a time. Due to time restrictions in the large survey, the decision was made to focus on adolescents’ issues with family management (e.g., influence in family decisions) and to assess views of mothers and fathers separately regarding individual reactions to children’s behaviors. Future studies should assess adolescents’ views on mothers’ and fathers’ overcontrol separately.

Despite its limitations, the current study has several strengths. First, we have used longitudinal data from a sample of early adolescents, who are generally underrepresented in the literature on social anxiety compared to children and adults [3], despite the fact that early adolescence is considered to be the time of onset for social anxiety [6]. We have followed adolescents and their parents over time for 2 years. Generally speaking, there is a need in the current literature to move beyond cross-sectional samples in order to understand processes over time, which this study has attempted to do. Finally, we have also included parents’ and adolescents’ ratings of their own behaviors, rather than just one or the other. Thus, this study contributes uniquely to the growing understanding about the links between adolescent social anxiety and parents’ overcontrolling behaviors.

### Summary

This study investigated the links between parental worry, over-control and adolescent social anxiety in a longitudinal sample of parent–adolescent dyads. Our results indicate that adolescents’ behavioral characteristics play an important role in predicting parents’ behaviors, such as their worries about their children. Social anxiety has many negative connotations, especially in the Western world, as adolescents with high levels of social fears have less involved social lives [54], and worse overall emotional adjustment [55] compared to adolescents without these issues. It is understandable that many parents worry about their adolescent children. The current results are an indication that social anxiety might invoke different reactions in terms of worries for mothers and fathers, respectively. Much remains to be done in a literature dominated by mother reports, and even our own data indicate that fathers aren’t as involved in research as mothers generally are. Nevertheless, for the fathers that did participate, findings indicate different links to adolescents’ social anxiety. Future studies should focus on what it is that fathers are doing exactly that differentiates them from mothers, in order to help abate adolescent social anxiety over time.
